# Effect of arm movement and task difficulty level on balance performance in healthy children: are there sex differences?

**DOI:** 10.1186/s13104-022-06195-w

**Published:** 2022-12-09

**Authors:** Thomas Muehlbauer, Mathew W. Hill, Simon Schedler

**Affiliations:** 1 Division of Movement and Training Sciences, Biomechanics of Sport, University of Duisburg-Essen, Gladbecker Str. 182, 45141 Essen, Germany; 2 Center for Sport, Exercise and Life Sciences, School of Life Sciences, Coventry University, Warwickshire, UK

**Keywords:** Postural control, Standing, Walking, Reaching, Upper extremities

## Abstract

**Objective:**

In children, studies have shown that balance performance is worse in boys compared to girls and further studies revealed inferior performance when arm movement was restricted during balance assessment. However, it remains unclear whether restriction of arm movement during balance testing differentially affects children’s balance performance (i.e., boys more than girls). Thus, we compared the influence of arm movement on balance performance in healthy boys versus girls (mean age: ~ 11.5 years) while performing balance tasks with various difficulty level.

**Results:**

In nearly all tests, balance performance (i.e., timed one-legged stance, 3-m beam walking backward step number, Lower Quarter Y-Balance test reach distance) was significantly worse during restricted compared to free arm movement but without any differences between sexes or varying levels of task difficulty. These findings indicated that balance performance is negatively affected by restriction of arm movement, but this does not seem to be additionally influenced by children’s sex and the level of task difficulty.

## Introduction

In childhood, the development of balance is characterized by a steady improvement [1], which is more pronounced in girls than in boys as reported in several studies [2–4]. Specifically, girls compared to boys showed less postural sway [2] and faster walking speed [3]. In addition to person-related factors such as sex, balance performance is also influenced by environmental and task-related factors [5]. One environmental factor is balance assessment procedure, as balance tasks can be performed with or without the use of arm movement. In the first case, the goal is to standardize the testing procedure, while in the second case, the aim is to obtain the maximal performance level. In fact, Hill et al. [6] showed that healthy children (mean age: 10.6 ± 0.5 years) achieved better balance performance (i.e., greater reach distances and shorter walking time) when the postural tasks were conducted with versus without the use of arm movement. In addition, it has been shown that task-related factors such as the level of difficulty also have an influence on balance performance. For example, Muehlbauer et al. [7] investigated healthy children (mean age: 11.5 ± 0.6 years) who performed the unipedal stance with a varying difficulty level. They reported greater performance differences between free versus restricted arm movement instruction during the higher (i.e., standing on foam ground with eyes closed) compared to the lower (i.e., standing on firm ground with eyes opened) difficulty condition.

In summary, previous literature [2, 3, 6, 7] showed that balance performance in children is influenced by person-, environment-, and task-related factors, with poorer performance detected for (i) boys versus girls [2, 3], (ii) restricted versus free arm movement [6, 7], and (iii) more versus less difficult postural task conditions [7]. However, to date, there is a lack of studies considering all three influencing factors within one study. Therefore, the aim of the present study was to investigate whether the use of arm movement has a differential influence on balance performance in children depending on sex and task difficulty level. We hypothesized that restricted arm movement will lead to poorer balance performance and this effect would be more pronounced in boys compared to girls and will increase with task difficulty level.

## Main text

### Methods

#### Participants

Forty children (18 boys, 22 girls) participated in this study (Table 1). There were no significant differences in the participants’ characteristics except for the maturity offset indicating that girls were more mature compared to boys. All subjects were healthy and free of any neurological or musculoskeletal impairments. None of the subjects had prior experience with the performed balance tests. Written informed consent and subject’s assent were obtained from all participants before the start of the study. Additionally, parent’s approval was obtained for minors.

**Table 1:** Characteristics of the study participants by sex

Characteristic	Boys (*n* = 18)	Girls (*n* = 22)	*p*-value
Age (years)	11.5 ± 0.6	11.5 ± 0.6	0.815
Maturity offset* (years from PHV)	– 1.6 ± 0.6	– 0.3 ± 0.7	< 0.001
Body mass (kg)	45.6 ± 14.6	42.8 ± 9.2	0.468
Body height (cm)	153.2 ± 8.5	152.3 ± 6.8	0.698
Body mass index (kg/m^2^)	19.0 ± 4.2	18.4 ± 3.2	0.557
Leg length (cm)	81.2 ± 5.4	79.2 ± 7.3	0.349

Data are presented as mean value ± standard deviation

*Maturity offset was calculated as years from peak height velocity (PHV) by using the formulas provided by Moore et al. [21]

#### Assessment of balance

Balance was determined by means of the One-Legged Stance (OLS) test. Participants stood without shoes on their dominant leg (i.e., kicking leg as determined per self-report). The participants were instructed to stand as long as possible but for a maximum of 60 s. The assessment was performed using four different levels of task difficulty: (1) standing with eyes opened on firm ground (EO, FI); (2) standing with eyes closed on firm ground (EC, FI); (3) standing with eyes opened on foam (i.e., AIREX Balance-pad) ground (EO, FO); (4) standing with eyes closed on foam ground (EC, FO). A total of two trials (one practice trial followed by one data-collection trial) were executed. The maximal stance time (sec) was used for further analysis. In children, the OLS test is a valid (concurrent and discriminative) and reliable (moderate to excellent) test for the assessment of balance performance [8, 9].

Balance was further assessed using the 3-m beam walking backward test [10]. The test consisted of wooden beams (length: 3 m; height: 5 cm) that differed in width (i.e., 6, 4.5, and 3 cm). The participants wore the same type of shoes (i.e., sports shoes) and were asked to walk backward at a self-selected speed from the beginning to the end of the beam but for a minimum of eight steps. A total of three trials (one practice trial followed by two data-collection trials) were performed. The number of steps for both data-collection trials per beam width was added up resulting in a maximum of 16 steps per beam and used for further analysis.

Lastly, balance was determined with the help of the Lower Quarter Y-Balance (YBT-LQ) test kit. The apparatus consisted of a central footplate to which three pipes were attached in the anterior (AT), posteromedial (PM), and posterolateral (PL) directions. Each pipe is marked in 1.0-cm increments for measurement purposes and was equipped with a moveable reach indicator block. Before testing, the respective length of the participants’ dominant leg was determined (i.e., distance in cm from the anterior superior iliac spine to the most distal aspect of the medial malleolus) [11]. Thereafter, participants were asked to reach with the non-dominant leg as far as possible in the AT, PM, and PL directions while standing with their dominant leg on the central footplate. The absolute maximal reach distance (cm) per reach direction was used for further analysis. In total, six trials (three practice trials followed by three data-collection trials) were executed. The normalized maximal reach distance (% leg length [LL]) per reach direction was calculated by dividing the absolute maximal reach distance (cm) by LL (cm) and then multiplying by 100. Further, the normalized (% LL) composite score (CS) was computed as the sum of the absolute maximal reach distance (cm) per reach direction divided by three times LL (cm) and then multiplied by 100.

#### Statistical analyses

Descriptive data are reported as group mean values and standard deviations. For all analyses, assumptions of normality (Shapiro–Wilk Test) and homogeneity of variance/sphericity (Mauchly Test) were checked and met prior to the application of analysis of variance (ANOVA). An arm × sex × task difficulty repeated measures ANOVA was conducted for the OLS test and the 3-m beam walking backward test. For the YBT-LQ test, an arm × sex repeated measures ANOVA was performed. In the case of significant differences, Bonferroni-adjusted post-hoc tests (i.e., paired *t*-tests) were performed. Further, effect size (*η*_p_^2^) was calculated and reported as small (0.02 ≤ *η*_p_^2^ ≤ 0.12), medium (0.13 ≤ *η*_p_^2^ ≤ 0.25), and large (*η*_p_^2^ ≥ 0.26) [12]. All statistical analyses were performed using Statistical Package for Social Sciences version 27.0 and the α-value was a priori set at *p* < 0.05 for all comparisons.

### Results

Table 2 displays balance performance with free compared to restricted arm movement by sex and Table 3 shows the main and interaction effects of the repeated measures ANOVA per outcome measure. A main effect of arm was observed for the most difficult stance condition (i.e., EC, FO) of the OLS test, for all three conditions of the 3-m beam walking backward test, and for all three reach directions as well as the CS of the YBT-LQ test. Post-hoc analyses revealed that balance performance was significantly better during free compared to restricted arm movement, irrespective of balance test considered. However, neither the main effect of sex nor the arm × sex or the arm × sex × task difficulty interactions reached the level of significance.

**Table 2:** Balance performance with free compared to restricted arm movement by sex

Test/Outcome	Boys (*n* = 18)		Girls (*n* = 22)	
	Free	Restricted	Free	Restricted
OLS test				
OLS time; EO, FI (s)	52.9 ± 14.0	54.8 ± 13.2	57.1 ± 15.7	51.4 ± 13.2
OLS time; EC, FI (s)	40.5 ± 26.2	37.6 ± 25.8	31.5 ± 22.1	31.7 ± 23.6
OLS time; EO, FO (s)	36.3 ± 23.6	34.3 ± 23.0	37.9 ± 22.6	32.0 ± 21.4
OLS time; EC, FO (s)	9.9 ± 7.8	6.9 ± 4.8	8.0 ± 5.2	5.0 ± 4.7
3-m beam walking backward test				
6-cm beam walk (steps)	13.3 ± 4.2	12.9 ± 4.2	14.0 ± 3.7	12.2 ± 4.1
4.5-cm beam walk (steps)	11.5 ± 5.2	10.4 ± 5.1	12.8 ± 3.7	10.2 ± 3.9
3-cm beam walk (steps)	10.2 ± 5.2	5.9 ± 3.3	8.2 ± 3.5	5.4 ± 3.2
YBT-LQ test				
YBT-LQ: AT reach (% LL)	79.3 ± 7.6	77.3 ± 7.8	77.1 ± 9.1	73.0 ± 9.7
YBT-LQ: PM reach (% LL)	113.1 ± 11.9	108.1 ± 12.1	109.0 ± 16.6	102.9 ± 13.6
YBT-LQ: PL reach (% LL)	108.3 ± 13.5	103.7 ± 13.6	105.8 ± 15.4	101.0 ± 13.1
YBT-LQ: CS (% LL)	100.2 ± 9.9	96.4 ± 10.3	97.3 ± 12.6	92.3 ± 11.1

Data are presented as mean value ± standard deviation

AT , anterior; CS , composite score; EC, eyes closed; EO, eyes opened; FI , firm ground; FO, foam ground; LL , leg length; OLS, One-Legged-Stance test; PL, posterolateral; PM, posteromedial; YBT-LQ , Lower Quarter Y Balance test

**Table 3:** Main and interaction effects of the repeated measures ANOVA per outcome measure

Test/Outcome	Main effects		Interaction effects	
	Arm	Sex	Arm × sex	Arm × sex × task difficulty
OLS test				
OLS time; EO, FI (s)	0.328 (0.03)	0.904 (0.01)	0.063 (0.09)	0.749 (0.01)
OLS time; EC, FI (s)	0.588 (0.01)	0.314 (0.03)	0.527 (0.01)	
OLS time; EO, FO (s)	0.209 (0.04)	0.954 (0.01)	0.542 (0.01)	
OLS time; EC, FO (s)	0.004 (0.20)*	0.216 (0.04)	0.977 (0.01)	
3-m beam walking backward test				
6-cm beam walk (steps)	0.052 (0.10)*	0.998 (0.01)	0.236 (0.04)	0.893 (0.01)
4.5-cm beam walk (steps)	0.006 (0.18)*	0.650 (0.01)	0.254 (0.03)	
3-cm beam walk (steps)	< 0.001 (0.44)*	0.249 (0.04)	0.268 (0.03)	
YBT-UQ test				
YBT-LQ: AT reach (% LL)	< 0.001 (0.36)*	0.237 (0.04)	0.108 (0.07)	–
YBT-LQ: PM reach (% LL)	< 0.001 (0.38)*	0.275 (0.03)	0.640 (0.01)	–
YBT-LQ: PL reach (% LL)	0.001 (0.26)*	0.544 (0.01)	0.947 (0.01)	–
YBT-LQ: CS (% LL)	< 0.001 (0.53)*	0.319 (0.03)	0.399 (0.02)	–

Values are *p*-values and effect sizes (*η*_p_^2^) in brackets. 0.02 ≤ *η*_p_^2^ ≤ 0.12 indicates small, 0.13 ≤ *η*_p_^2^ ≤ 0.25 indicates medium, and *η*_p_^2^ ≥ 0.26 indicates large effects

AT, anterior; CS, composite score; EC, eyes closed; EO, eyes opened; FI, firm ground; FO, foam ground; LL, leg length; OLS, One-Legged-Stance test; PL, posterolateral; PM, posteromedial; YBT-LQ, Lower Quarter Y Balance test

*Indicates significant (*p* < .0.05) performance difference in favor of free arm movement

### Discussion

In the present study, we compared the influence of arm movement on balance performance in healthy boys versus girls that performed postural tasks with various difficulty level. In almost all test conditions, significantly worse balance performance was observed under restricted compared to free arm movement test conditions (Tables 2 and 3). This finding is in accordance to our assumption and corresponds with those from previous studies that also detected a negative effect of arm movement restriction on balance performance. For example, Objero et al. [13] reported more postural sway during standing with restricted arm movement. Further, Hill et al. [6] detected longer times to walk a limited distance of two meters while arm movement was restricted. Lastly, Hébert-Losier et al. [14] found shorter YBT-LQ reach distances when arms were fixed on the hips. Only in the less difficult stance test conditions, no significant influence of arm movement was found, which can most likely be explained by a “ceiling effect”. In summary, it can be deduced that environmental factors like arm movement should be allowed if the goal is to detect better balance performance values. In this regard, previous literature [6, 15, 16] showed that allowing free arm movements has a positive effect on the mechanical aspects of the body by (i) increasing the moment of inertia, (ii) acting as a counterweight to shift the centre of mass away from the direction of instability, or (iii) generating a reactive torque to counteract the whole-body angular momentum.

Contrary to our expectation, the negative effect of restricted arm movement on balance performance was not additionally influenced by children’s sex (Table 3). In this regard, original studies [2, 3] and a systematic review article [4] reported poorer balance performance in boys compared to girls, so a greater negative effect was hypothesized for the former when arm movement was restricted. The reason given is that in childhood the postural control system of boys is less mature (e.g., central nervous structures) than that of same aged girls [17, 18]. In fact, in the present study, boys had the same chronological age but showed a significantly lower maturity offset compared to girls (Table 1). However, we could not find any arm by sex interaction, which suggests that no specific consideration of this person-related factor is necessary during balance testing in children.

Further, the negative effect of arm movement restriction on balance performance did not increase with increasing task difficulty (Table 3). This finding is contrary to our assumption as well and different from former studies [13, 19, 20] that found a greater effect of arm movement restriction in more compared to less difficult postural tasks. Most likely, the discrepancy between the applied difficulty levels was too small to have an additional negative effect on balance performance besides arm movement restriction. To validate this explanation, balance tasks with larger discrepancies in terms of the task-related factor difficulty level should be used in future studies.

## Conclusion

The present study compared the effect of arm movement on balance performance between healthy male and female children while performing postural tasks with various difficulty level. Restricted versus free arm movement yielded worse balance performance, irrespective of sex and task difficulty. In healthy children, these findings indicate that arm movements during balance assessment rather than sex and task difficulty level are an important environmental impact factor. Thus, descriptions on arm positioning during balance assessment are necessary to facilitate data replication.

## Limitations

- Only healthy children were studied, which prevents the transfer of our findings to children with balance problems (e.g., due to injuries).
- Well-established field tests (i.e., OLS test, 3-m beam walking backward test, YBT-LQ test) but no instrumented biomechanical procedures (e.g., postural sway via force plate) were used.
- Performance data during standing, walking, and reaching were collected but no kinematic data (e.g., using a motion capture system) of arm movements were registered.

## Data Availability

The data generated and analyzed during the present study are not publicly available due to ethical restrictions but are available from the corresponding author upon reasonable request.

## Abbreviations

ANOVA: Analysis of variance
AT: Anterior
CS: Composite score
EC: Eyes closed
EO: Eyes opened
FI: Firm ground
FO: Foam ground
LL: Leg length
OLS: One-Legged Stance test
PL: Posterolateral
PM: Posteromedial
YBT-LQ: Lower Quarter Y Balance test
